# Histological classifi cation of 1,025 cases of Hodgkin's lymphoma from the State of São Paulo, Brazil

**DOI:** 10.1590/S1516-31802005000300009

**Published:** 2005-05-02

**Authors:** José Vassallo, Roberto Pinto Paes, Fernando Augusto Soares, Yara Menezes, Vera Aldred, Karina de Cássia Braga Ribeiro, Antonio Correa Alves

**Keywords:** Hodgkin's disease, Pathology, Classification, Lymphoma, Neoplasms, Doença de Hodgkin, Patologia, Classificação, Linfoma maligno, Neoplasias

## Abstract

**CONTEXT AND OBJECTIVE:**

It is currently asserted that, in industrialized countries, nodular sclerosis is the most frequent type of Hodgkin's lymphoma, in contrast to developing countries, where mixed cellularity and lymphocyte depletion are more frequently seen. The objective was to review histological data from cases of Hodgkin's lymphoma from São Paulo and Campinas cities.

**DESIGN AND SETTING:**

Cross-sectional histopathological analysis, in four university hospitals and one cancer care center.

**METHODS:**

1,025 cases diagnosed as Hodgkin's lymphoma between 1990 and 2000 were collected from five institutions; 631 of them (61.5%) had been immunophenotyped using antibodies to CD20, CD3, CD15 and CD30. The relative frequencies of histological types (as informed by the contributing authors, who are hematopathologists in their institutions) were determined according to age and gender.

**RESULTS:**

The Hodgkin's lymphoma types were distributed as follows: lymphocyte predominance 4.8%, nodular sclerosis 69.2%, mixed cellularity 21.1% and lymphocyte depletion 4.6%.

**CONCLUSIONS:**

The controversy regarding the frequencies of Hodgkin's lymphoma types within the Brazilian setting seems to be due to the small number of cases in previous studies. The present data show a picture close to the situation in the industrialized countries.

## RESUMO

**CONTEXTO E OBJETIVO:**

Tem-se afirmado correntemente que, em países industrializados, a esclerose nodular é o tipo mais freqüente de linfoma de Hodgkin, ao contrário de países em desenvolvimento, onde a celularidade mista e a depleção linfocitária são mais freqüentes. O objetivo do estudo é rever os dados histológicos de linfoma de Hodgkin das cidades de São Paulo e Campinas.

**TIPO DE ESTUDO E LOCAL:**

Transversal, por análise histopatológica, em quatro hospitais universitários e um centro oncológico de referência.

**MÉTODOS:**

1.025 casos com o diagnóstico de linfoma de Hodgkin entre 1990 e 2000 foram coletados de cinco instituições. Em 631 (61,5%) casos fora feito estudo imunoistoquímico para os marcadores CD20, CD3, CD15 e CD30. As freqüências relativas dos tipos histológicos (informadas pelos autores que são hematopatologistas de suas respectivas instituições) foram determinadas nos diversos grupos etários e por gênero.

**RESULTADOS:**

Os tipos de linfoma de Hodgkin foram assim distribuídos: predominância linfocitária 4,8%, esclerose nodular 69,2%, celularidade mista 21,1% e depleção linfocitária 4,6%.

**CONCLUSÕES:**

Dados controversos sobre a freqüência dos tipos de linfoma de Hodgkin em nosso meio parecem ser devidos ao pequeno número de casos dos trabalhos anteriores. Nossos dados são comparáveis aos dos países industrializados.

## INTRODUCTION

In 1973, Correa and O'Conor studied the geographical pathology of lymphoreticular tumors, among which 147 cases of Hodgkin's lymphoma (HL) were included. These consisted of 97 cases originating from Recife, in northeastern Brazil, of lower socioeconomic level, and 50 from São Paulo, in southeastern Brazil, a more industrialized region of this country. They found that the HL cases were predominantly among males, especially those under 20 years of age, an age group in which the male/female ratio was 3.7. In that study, the nodular sclerosis (NS) and lymphocyte predominance (LP) types predominated in Recife (ratio NS + LP/MC + LD = 1.4 for males and 1.7 for females), while the mixed cellularity (MC) and lymphocyte depletion (LD) types predominated in São Paulo (ratio NS + LP/MC + LD = 0.59 for males and 0.81 for females).^1^

Later studies showed somewhat different data on the distribution of histological types of HL. Faria et al.^2^ studied 36 patients aged under 17 years who were diagnosed in Campinas, State of São Paulo, between 1978 and 1988. They found 19 cases (52.7%) of NS, 14 (38.8%) MC, and three (8.3%) LD. In the same city, in a study of 134 patients over 15 years of age diagnosed between 1985 and 1994, de Souza et al.^3^ found 7 cases (5.2%) of LP, 67 (50.4%) NS, 46 (34.6%) MC, 12 (9%) LD and two unclassified. Spector et al.^4,5^ reported 54% NS, 36% MC, 8% LP and 2% LD among 59 patients from the city of Rio de Janeiro, also located in southeastern Brazil. The same group, studying 51 patients in 2002,^6^ found an even higher frequency for the NS type (65%). The predominance of the NS type of HL in these studies approached the frequency reported from industrialized countries, although the frequency of Epstein-Barr virus-related HL in Brazil is similar to what has been found in other developing countries.^7^

In a recent study of 96 cases by Elgui de Oliveira et al.,^8^ predominance of the MC type of HL was seen among children in northeastern and southeastern Brazil and among adults in the southeast. Among adults in the northeast, the NS type was more frequent.

In view of the controversy regarding the data, and as all these studies were based on small numbers of cases, the present authors, who are members of the hematopathology committee of the Brazilian Society of Pathology, proposed a multicenter study of the histological types of HL from five institutions in the State of São Paulo, Brazil.

## METHODS

A total of 1,025 cases diagnosed between 1990 and 2000 were collected from the five institutions. Four of these institutions are university hospitals and one is an oncology center. Four of them are located in the city of São Paulo and one in Campinas, which are the two largest cities in the state of São Paulo. These hospitals are public or non-profit and mainly care for patients from the lowest socioeconomic levels. The cases were distributed as follows: 276 from the School of Medicine of Santa Casa de Misericórdia de São Paulo, 255 from Hospital do Câncer de São Paulo (Fundação Antônio Prudente), 187 from the Hospital das Clínicas da Universidade de São Paulo, 169 from the Hospital das Clínicas da Universidade Estadual de Campinas, and 138 from Universidade Federal de São Paulo — Escola Paulista de Medicina. For some of the cases (631 or 61.5%), immunophenotyping was available, which had been done using antibodies to CD20, CD3, CD15 and CD30. It was available for 190 cases (68.8%) from Santa Casa de Misericórdia de São Paulo, all cases from Hospital do Câncer, 74 (39.5%) from Hospital das Clínicas da Universidade de São Paulo, 87 (51.4%) from Universidade de Campinas and 25 (18.1%) from Universidade Federal de São Paulo.

The final diagnosis was established by the hematopathology specialists of each institution who are the authors of the present study. The original diagnoses were only changed by the local experts in a few cases in the institutions, in which the diagnosis had been made by other pathologists. At the Hospital do Câncer de São Paulo, out of the initial 283 cases selected and submitted to immunophenotyping, 255 diagnosis of Hodgkin's lymphoma were maintained. In 28 cases the diagnosis was changed: in thirteen cases to diffuse large B-cell non-Hodgkin's lymphoma, in ten to T-cell-rich large B-cell lymphoma, in three to anaplastic large cell lymphoma and in two to peripheral T-cell lymphoma. At Universidade Federal de São Paulo, 18 cases had a change in sub-typing of Hodgkin's lymphoma: twelve from mixed cellularity to nodular sclerosis and six from nodular sclerosis to mixed cellularity. In all the other institutions, all the original diagnoses were maintained.

The statistical analysis consisted of estimation of absolute and relative frequencies, and utilization of the chi-squared test to verify associations between category variables. For all tests, the alpha error was established as 5%.

## RESULTS

Six hundred and seventeen patients (60.2%) were male and 408 (39.8%) female. The age distribution was as follows: 88 patients (8.5%) were in their first decade of life, 235 (22.9%) in the second, 276 (26.9%) in the third, 159 (15.5%) in the fourth, 105 (10.2%) in the fifth, 70 (6.8%) in the sixth, 47 (4.5%) in the seventh and 45 (4.3%) in the eighth or older. Fourteen patients were five years old or less and 8 were over 80 years old. The histological classification of HL was: 710 (69.2%) NS, 217 (21.1%) MC, 50 (4.8%) LP and 48 (4.6%) LD. Table 1 summarizes the data from the patients studied. Table 2 shows the distribution of histological types of HL in each institution.

**Table 1:** Hodgkin's lymphoma in 1,025 cases from five institutions in Brazil, according to histological types, age and gender

	LP		NS		MC		LD		
Age	M	F	M	F	M	F	M	F	Total
0-10	9 (10.2)	1 (1.1)	32 (36.3)	15 (17)	22 (25)	5 (5.6)	3 (3.4)	1 (1.1)	88
**10-20**	7 (2.9)	4 (1.7)	95 (40.4)	83 (35.3)	28 (11.9)	9 (3.8)	5 (2.1)	4 (1.7)	**235**
**20-30**	4 (1.4)	2 (0.7)	103 (37.3)	112 (40.5)	28 (10.1)	14 (5)	6 (2.1)	7 (2.5)	**276**
**30-40**	7 (4.4)	1 (0.6)	70 (44)	37 (23.2)	23 (14.4)	12 (7.5)	6 (3.7)	3 (1.8)	**159**
**40-50**	4 (3.8)	2 (1.9)	37 (35.2)	28 (26.6)	22 (20.9)	5 (4.7)	5 (4.7)	2 (1.9)	**105**
**50-60**	2 (2.8)	1 (1.4)	27 (38.5)	16 (22.8)	17 (24.2)	6 (8.5)	0	1 (1.4)	**70**
**60-70**	2 (4.2)	1 (2.1)	18 (38.2)	12 (25.5)	4 (8.5)	6 (12.7)	3 (6.3)	1 (2.1)	**47**
**> 70**	1 (0.2)	2 (0.4)	19 (42.2)	6 (13.3)	8 (17.7)	8 (17.7)	0	1 (0.2)	**45**
**Total**	**36 (3.5)**	**14 (1.3)**	**401 (39.1)**	**309 (30.1)**	**152 (14.8)**	**65 (6.3)**	**28 (2.7)**	**20 (1.9)**	**1,025**

*LP = lymphocyte predominance; NS = nodular sclerosis; MC = mixed cellularity; LD = lymphocyte depletion; M = males; F = females; numbers in parenthesis = percentage of the total for the same age group.*

**Table 2:** Distribution of histological types of Hodgkin's lymphoma in each of five Brazilian institution (n = 1,025)

Institutions	Nodular sclerosis	Mixed cellularity	Lymphocyte predominance	Lymphocyte depletion	Total
**Santa Casa de Misericórdia**	225 (81.5%)	43 (15.5%)	6 (2.1%)	2 (0.7%)	**276**
**Hospital do Câncer**	201 (82.3%)	36 (14.1%)	15 (5.8%)	3 (1.1%)	**255**
**Universidade de São Paulo**	73 (39%)	74 (39.5%)	10 (5.3%)	30 (16%)	**187**
**Universidade Estadual de Campinas**	113 (66.8%)	45 (26.6%)	4 (2.3%)	7 (4.1%)	**169**
**UniversidadeFederal de SãoPaulo**	98 (71%)	19 (13.7%)	15 (10.8%)	6 (4.3%)	**138**
**Total**	**710 (69.2%)**	**217 (21.1%)**	**50 (4.8%)**	**48 (4.6%)**	**1,025**

There was a statistically significant association between gender and histological type, with a higher frequency of NS among women (p = 0.001) (Table 3). No significant association between gender and age group was noted (p = 0.242) (Table 4). A higher proportion of patients in their first or second decade of life were found at Hospital do Câncer de São Paulo (42.7%).

**Table 3:** Patient distribution according to gender and histological type in 1,025 of Hodgkin's lymphoma in five institutions in Brazil

	Histological type (%)			
Gender	NS	LP	MC	LD
**Male**	401 (65.0)	36 (5.8)	152 (24.7)	28 (4.5)
**Female**	309 (75.7)	14 (3.4)	65 (16.0)	20 (4.9)
**Total**	**710 (69.3)**	**50 (4.9)**	**217 (21.2)**	**48 (4.6)**

*χ^2^ = 15.86, p = 0.001; LP = lymphocyte predominance; NS = nodular sclerosis; MC = mixed cellularity; LD = lymphocyte depletion.*

**Table 4:** Patient distribution according to gender and age group in 1,025 of Hodgkin's lymphoma in five institutions in Brazil

	Age group (%)			
Gender	0-19 years	20-39 years	40-59 years	≥ 60 years
**Male**	201 (32.6)	252 (40.8)	114 (18.5)	50 (8.1)
**Female**	122 (29.9)	188 (46.1)	61 (14.9)	37 (9.1)
**Total**	**323 (31.5)**	**440 (42.9)**	**175 (17.1)**	**87 (8.5)**

*χ^2^ = 4.18, p = 0.242.*

## DISCUSSION

There was overall male predominance (male/female ratio = 1.5) among our patients. This ratio was higher for the LP and MC types (2.5 and 2.3, respectively) and lower for the NS and LD types (1.2 and 1.4, respectively). Predominance of 70% males for the MC type is in accordance with data from the World Health Organization Classification of Tumors publication, but the findings for the LD type differ.^9^ The age distribution of the present study, showing a maximum of cases in the third decade (26.9%) and a decline thereafter, was also seen in an American study of 35,033 patients.^10^ However, our study found that the proportion of patients in their first two decades was 31.5%, while this corresponded to 13% in the American study. Correa and O'Conor^1^ had also reported a high proportion of cases of HL among children and young adults in Brazil in their study.

The frequency of HL types was similar to what has been reported from developed countries. In the study by Kennedy et al.^10^ there was 60.8% NS, 19.1% MC, 6.3% LP and 2.2% LD. The discrepancies reported in previous studies in Brazil were probably due to sampling problems, since the diagnosis of HL subtypes can be considered reliable, at least for the NS type.^11^

The current study presents for the first time data from a large number of cases of HL in Brazil. It confirms the predominance of the NS type and the high proportion of patients in their first two decades of life.

## CONCLUSION

The controversy regarding the frequencies of Hodgkin's lymphoma types within the Brazilian setting seems to be due to the small number of cases in previous studies. The present data present a picture close to the situation in the industrialized countries.
